# Genes and pathways for CO_2 _fixation in the obligate, chemolithoautotrophic acidophile, *Acidithiobacillus ferrooxidans*, Carbon fixation in *A. ferrooxidans*

**DOI:** 10.1186/1471-2180-10-229

**Published:** 2010-08-27

**Authors:** Mario Esparza, Juan Pablo Cárdenas, Botho Bowien, Eugenia Jedlicki, David S Holmes

**Affiliations:** 1 Center for Bioinformatics and Genome Biology, MIFAB, Fundación Ciencia para la Vida and Depto. de Ciencias Biologicas, Facultad de Ciencias Biologicas, Universidad Andres Bello, Santiago, Chile; 2 ICBM, Faculty of Medicine, University of Chile, Santiago, Chile; 3 Institut für Mikrobiologie und Genetik, Georg-August-Universität Göttingen, Göttingen, Germany; 4 Departamento de Acuicultura, Facultad de Recursos del Mar, Universidad de Antofagasta, Antofagasta, Chile

## Abstract

**Background:**

*Acidithiobacillus ferrooxidans *is chemolithoautotrophic γ-proteobacterium that thrives at extremely low pH (pH 1-2). Although a substantial amount of information is available regarding CO_2 _uptake and fixation in a variety of facultative autotrophs, less is known about the processes in obligate autotrophs, especially those living in extremely acidic conditions, prompting the present study.

**Results:**

Four gene clusters (termed *cbb1-4*) in the *A. ferrooxidans *genome are predicted to encode enzymes and structural proteins involved in carbon assimilation via the Calvin-Benson-Bassham (CBB) cycle including form I of ribulose-1,5-bisphosphate carboxylase/oxygenase (RubisCO, EC 4.1.1.39) and the CO_2_-concentrating carboxysomes. RT-PCR experiments demonstrated that each gene cluster is a single transcriptional unit and thus is an operon. Operon *cbb1 *is divergently transcribed from a gene, *cbbR*, encoding the LysR-type transcriptional regulator CbbR that has been shown in many organisms to regulate the expression of RubisCO genes. Sigma^70^-like -10 and -35 promoter boxes and potential CbbR-binding sites (T-N_11_-A/TNA-N_7_TNA) were predicted in the upstream regions of the four operons. Electrophoretic mobility shift assays (EMSAs) confirmed that purified CbbR is able to bind to the upstream regions of the *cbb1*, *cbb2 *and *cbb3 *operons, demonstrating that the predicted CbbR-binding sites are functional *in vitro*. However, CbbR failed to bind the upstream region of the *cbb4 *operon that contains *cbbP*, encoding phosphoribulokinase (EC 2.7.1.19). Thus, other factors not present in the assay may be required for binding or the region lacks a functional CbbR-binding site. The *cbb3 *operon contains genes predicted to encode anthranilate synthase components I and II, catalyzing the formation of anthranilate and pyruvate from chorismate. This suggests a novel regulatory connection between CO_2 _fixation and tryptophan biosynthesis. The presence of a form II RubisCO could promote the ability of *A. ferrooxidans *to fix CO_2 _at different concentrations of CO_2_.

**Conclusions:**

*A. ferrooxidans *has features of *cbb *gene organization for CO_2_-assimilating functions that are characteristic of obligate chemolithoautotrophs and distinguish this group from facultative autotrophs. The most conspicuous difference is a separate operon for the *cbbP *gene. It is hypothesized that this organization may provide greater flexibility in the regulation of expression of genes involved in inorganic carbon assimilation.

## Background

*Acidithiobacillus ferrooxidans *is a mesophilic, obligately chemolithoautotrophic, γ-proteobacterium that gains energy and reducing power from the oxidation of ferrous iron and reduced inorganic sulfur compounds (RISCs) [1]. It grows optimally at pH 2, although growth as low as pH 1 has been reported [2]. The microorganism is a key player in the solubilization of copper in industrial bioleaching operations and makes an important contribution to the biogeochemical cycling of nutrients and metals in pristine and manmade acidic environments. In such environments, CO_2 _would be expected to exist preferentially as a dissolved gas in equilibrium with the atmosphere and not in the bicarbonate form typically found at circum-neutral pHs [3].

*A. ferrooxidans *has previously been shown [4,5] to have candidate genes (*cbbL *and *cbbS*) for the large and small subunits of ribulose-1,5-bisphosphate carboxylase/oxygenase (RubisCO, EC 4.1.1.39) that catalyses CO_2 _fixation by the Calvin-Benson-Bassham (CBB) cycle in many organisms [6]. *cbbL *and *cbbS *are linked to genes predicted to encode carboxysome shell proteins [7] and a divergently transcribed gene encoding the LysR-type transcription regulator CbbR [4]. The intergenic region between *cbbR *and *cbbL *is predicted to harbor binding sites for CbbR [4]. In addition, microarray transcript profiling experiments have detected differential expression of several genes in *A. ferrooxidans *potentially involved in the CBB cycle depending on the growth substrate used [8].

These observations taken together, suggest that, in *A. ferrooxidans*, CbbR can regulate the expression of RubisCO and the carboxysome genes and therefore is likely to be involved in the regulation of carbon fixation as has been observed in other autotrophic bacteria including: *Xanthobacter flavus *[9], *Ralstonia eutropha *H16 [10], *Chromatium vinosum *[11], *Nitrobacter vulgaris *[12], *Halothiobacillus neapolitanus *[13], *Thiobacillus denitrificans *[14], *Rhodobacter sphaeroides *[15], *Rhodobacter capsulatus *[16], *Rhodospirillum rubrum *[17], *Hydrogenovibrio marinus *[18], *Nitrosomonas europaea *[19] and *Thiomicrospira crunogena *XCL-2 [20]. However, no coherent model has been developed for *A. ferrooxidans *to explain all the data and little experimental evidence has been provided to support several of the aforementioned observations, prompting the current investigation.

## Methods

### Bacterial strains and culture conditions

Information regarding bacterial strains and plasmids used in this study is provided in Table 1. *A. ferrooxidans *was cultured in 9 K medium (adjusted to pH 3.5 with H_2_SO_4_) containing 5 g/l elemental sulfur at 30°C under aerobic conditions on a rotary shaker at 150 rpm as described previously [21]. *Escherichia coli *harboring plasmids was grown at 37°C in LB broth with ampicillin (Amp: 100 μg/ml).

**Table 1:** List of bacterial strains and plasmids used in this study

Strain or plasmid	Relevant characteristic	Source or reference
**Bacterial strains**		
*Acidithiobacillus* *ferrooxidans*	Type strain	ATCC 23270
*E. coli *TOP10	F^- ^*mcr*A Δ(*mrr*-*hsd*RMS-*mcr*BC) ϕ80*lac*ZΔM15 Δ*lac*X74 *rec*A1 a*ra*D139 Δ(*ara-leu*) 7697 *gal*U *gal*K *rps*L (Str^R^) *end*A1 *nup*G	Invitrogen
**Plasmids**		
pBAD-TOPO^®^	Amp^R ^promoter *ara*BAD (P_BAD_) C-terminal: V5 epitope tag-polyhistidine (6 × His)	Invitrogen
pBAD-*cbb*R	pBAD-TOPO::927-bp fragment containing *cbb*R from *A. ferrooxidans *ATCC 23270 expressed from P_BAD _promoter	This study

Abbreviations used: ATCC, American Type Culture Collection. Amp^R^, ampicillin resistance; Str^R^, streptomycin resistance.

### General DNA techniques and sequencing of DNA

*A. ferrooxidans *cultures were centrifuged at 800 × g to remove solid sulfur precipitates prior to cell harvest. Unattached cells were pelleted at 8000 × g for 10 min. The cell pellet was resuspended in 9 K salt solution for washing and washed cells were collected by centrifugation at 8000 × g for 10 min as described previously [21]. Standard procedures [22] were employed to isolate genomic and plasmid DNA from bacteria, to transform plasmid DNA into *E. coli*, and for general DNA handling. Restriction endonucleases and DNA-modifying enzymes were used as recommended by the manufacturers. Plasmid DNA was prepared by means of the QIAprep Spin Mini Kit (Qiagen). Polymerase chain reaction (PCR) products were amplified with Taq DNA polymerase (Fermentas) and purified from agarose gels using the QiaEx DNA Purification Kit (Qiagen). Each PCR reaction contained in a volume of 25 μl 1 ng of template DNA, 0.5 μM of required primers and 0.2 mM of each deoxyribonucleotide in 1× PCR buffer containing 1.5 mM MgCl_2 _(Fermentas). PCR conditions were as follows: initial denaturing step at 95°C for 5 min followed by 30 amplification cycles (denaturation at 95°C for 30 s, annealing at the appropriate temperature depending on the specific primer pairs for 20 s, elongation at 72°C) and a final elongation step at 72°C for 10 min. DNA sequencing of pBAD-*cbbR *was carried out by the Göttingen Genomics Laboratory (Göttingen, Germany).

### Isolation of RNA and RT-PCR

Total RNA was isolated from cells of *A. ferrooxidans *grown to mid-log phase in 9 K medium supplemented with sulfur, as described previously [23]. The RNA preparation was treated with DNase I (Fermentas) before proceeding with the cDNA synthesis step. One microgram of total cellular RNA was used for each reaction. Reverse transcription-PCR (RT-PCR) was performed on purified RNA using the One-Step RT-PCR kit (Qiagen). The sequences of the RT and PCR primers used are provided in Table 2. As controls, reactions were carried out that included RNA but lacked reverse transcriptase to assess genomic DNA contamination and that lacked RNA but contained 1 ng of genomic DNA.

**Table 2:** Sequences of primers used in co-transcription RT-PCR assays, cloning experiments and EMSA assays.

Primers used in RT-PCR assays					
***cbb1 *operon**					

Number^a^	Gene	Forward primer (5'-3')	Number^a^	Gene	Reverse primer (5'-3')

1	*cbbR*	CAACGCCGTGTTGCTCGAA	2	*cbbL1*	CTAGACTTTTTTACGGCCATGCTT
3	*cbbL1*	CTGCCAATCGTGTCGCGC	4	*csoS2*	CGCACGGGAAAGCGACTT
5	*csoS2*	CCTATGGTGCCGTGCCAAC	6	*csoS3*	GTGCATGACGCACGCCC
7	*csoS3*	GTCAGCGGGTCAAAGCCG	8	*csoS1A*	GCCGCCTTGGTCATCG
9	*csoS1B*	GGAGCAGATGCGTGTGAGCG	11	*parA*	AGTAGAACCCCGCCGAGCCAA
10	*bfrA*	CGCGCAGAAGAGTTACAAGCCTTG	12	*parA*	CTGATCGAACCCTGAGGATCGG
13	*parA*	GTGCTGCGGTTGAAGGGGT	14	*hyp2*	GTGGAGTTCGATAATGGCGGAG
15	*hyp2*	CGAGAAGCCTCCGCCATTATC	16	*cbbQ1*	GCCTGTGGGTCCTTTCAGCAT
18	*cbbO1*	TGACGCCAGGAAAGCGGTG	17	*cbbO1*	CAGGGATTTCAGGCTGGTCG
19	*cbbO1*	GCAGAGGCTGCCAGAAAAGCT	20	*cbbA*	AAGCACCTACCGCGTATCCGT
			21	*bioD^a^*	CAGTGCCACCGCCACCC

***cbb2 *operon**					

	Forward primer (5'-3')'		Reverse primer (5'-3')		

1	*tatC^a^*	ACGACGGCGTCTAGAACCGCC	2	*cbbL2*	CCGGTAATCCTCTAGACCCGCGTT
3	*cbbL2*	CATCGAGAAGGAAGGCAAGGC	4	*cbbS2*	CGCAACCTGTTGACGGATCTG
5	*cbbS2*	ACCGGAAAACGCCTTCGGC	6	*cbbQ2*	GGTCAATGGGCCATCCTGCC
7	*cbbQ2*	AGGGTGTTGAGGCGAAGGCC	8	*cbbO2*	GTACGATGGGCGTGTGCGC
9	*cbbO2*	GCCTACAGCGAGGAGGCCATG	10	*yfeA^a^*	GCGGAGCCTTGTCCCTCGG

***cbb3 *operon**					

		Forward primer (5'-3')'			Reverse primer (5'-3')

1	*hyp4^a^*	TACGAAGGCGGCTCCCCG	2	*hyp3*	CGACGGCAATCGGAGTCTTT
3	*hyp3*	CGGGTGATCGCGCTGGAT	4	*cbbT*	CAGAATGCCGTCGTGACCA
5	*cbbT*	ATCGGCATCGACCACTTT	6	*cbbK*	TCCATCATACGCAGGACA
7	*cbbK*	CCTACATCAGTACGGGTG	9	*cbbA*	CACCTGCTCCAGGTTGTT
8	*pykA*	TTGATCCTCATCACCATCGG	11	*cbbE*	GATATGGATATAGTCGGCACCC
10	*cbbA*	GCAGGCCAGCAAGATCAA	14	*trpE*	GCCGACAAGGGAGTATCGA
12	*cbbE*	CTATCGAACTGGAAGTGGATGG	16	*trpG*	CGATAGCCGCCACGTCG
13	*cbbZ*	TCGGCGATTCACGTAACG	17	*trpC^a^*	AGGGCCACTGCCGCCTGC
15	*trpE*	GAAACCATGAACAAACGCCG			

***cbb4 *operon**					

		Forward primer (5'-3')'			Reverse primer (5'-3')

1	*ompA^a^*	GGTATTTCCTATTTTTGGGGTGGC	3	*sahA*	CGGCAATGCGGACTTCCTTAC
2	*metK*	TTGGGAGCGGACCGACAAG	5	*metF*	AAGCATACTCGGGACCCAAGG
4	*sahA*	CTTCGCGGGGGTGCTGA	7	*cbbP*	GACGGGATGTTTTTTGGACATGG
6	*metF*	CACCGAGCCTGCATTTTTACACC	9	*ynbD*	GGCTACAGCCACCACGGGAT
8	*cbbP*	ATGTTGCCGGGCAGTTTTATGTC	10	*fbnA^a^*	GCGAGGTGGACTGGACGGA

**Primers used in EMSA assays and cloning experiments**					

Letter designation		Forward primer (5'-3')			Reverse primer (5'-3')

(a) Pcbb1		CGGCAGCGAAGATCTTGAGTTGGTGC	(b) Pcbb1		CTCCGGCCTCATACTTTTTTACGG
(c) *cbbRfw*		TCTATCCGTCATGCAACCTTG	(d) *cbbRrev*		GCGCCATTCCTTTTCACCATG
(e) Pcbb2		ACGACGGCGGCAAGCACCGCC	(f) Pcbb2		CCGGTAATCCTTCACACCCGCGTT
(g) Pcbb3		CATTGAACAGGGTCAGCTCCTGG	(h) Pcbb3		ATCGGAGTCTTTGATCATGCGCC
(i) Pcbb4		TTTGGGGTGGCAGCAAGAAGT	(j) Pcbb4		GGAAACGGATTCAGAGGTGAAAAG

^a^Genes lie outside the operons and are not shown in Figure 2.

### Cloning and expression of *cbbR*

A DNA fragment corresponding to the coding region of *cbbR *of *A. ferrooxidans *was amplified by PCR using primers (Integrated DNA Technologies) *cbbRfw *and *cbbRrev *(Table 2). The amplified product was cloned into the expression vector pBAD-TOPO (Invitrogen) according to the manufacturer's instruction. The resulting plasmid pBAD-*cbbR *was introduced by electroporation into *E. coli *TOP10 (Invitrogen) competent cells [22]. *E. coli *was grown at 37°C in 10 ml LB containing 100 μg/ml ampicillin to an OD_600 _of 0.8. Overproduction of the recombinant His_6_-tagged CbbR protein was initiated by adding arabinose to a final concentration of 0.1% (w/v) with continued shaking at 200 rpm for 12 h.

### Purification of CbbR

Cells from 1.5 l of induced culture were harvested by centrifugation (8,000 × g for 10 min at 4°C) and at -20°C. After thawing the cell pellet was resuspended in 40 ml denaturing buffer containing 6 M guanidine-HCl, 100 mM NaH_2_PO_4 _and 10 mM Tris-HCl, pH 8.0, and incubated at room temperature with continuous stirring for about 30 min until inclusion body proteins were solubilized. Any remaining insoluble material was removed by centrifugation at 18,000 × g and 7°C for 20 min. The resulting supernatant was filtered through a 0.45-μm membrane and the recombinant protein subsequently purified by affinity chromatography on a 2.5-ml Ni-nitrilotriacetic acid column under amalgam conditions (denaturing conditions-native conditions). Initially the protein was adsorbed to the matrix under denaturing conditions at room temperature after equilibration with binding denaturing buffer (BDB) containing 8 M urea, 100 mM NaH_2_PO_4 _and 10 mM Tris-HCl, pH 8.0. The column was first washed with BDB and then with washing denaturing buffer (WDB) containing 8 M urea, 100 mM NaH_2_PO_4_, 10 mM Tris-HCl, pH 6.0. The elution of CbbR was carried out under native conditions at 4°C after equilibrating the column with native buffer (20 mM imidazole, 300 mM NaCl, 50 mM NaH_2_PO_4_, pH 8.0). His_6_-CbbR was eluted at a flow rate of 1 ml/min with eluting native buffer (250 mM imidazole, 300 mM NaCl, 50 mM NaH_2_PO_4_, pH 8.0). The eluted fractions were monitored at 280 nm. Fractions with the highest protein content were pooled, dialysed twice against 50 mM HEPES-NaOH, pH 7.8 containing 200 mM KCl, 10 m MgCl_2_, 1 mM dithiothreitol, 0.05 mM phenylmethylsulfonyl fluoride and 50% (w/v) glycerol. The final protein concentration was 4 mg/ml. Protein preparations were analyzed by SDS-polyacrylamide gel electrophoresis in 12% (w/v) polyacrylamide slab gels under reducing conditions in the presence of 100 mM β-mercaptoethanol. Gels were stained with Coomassie Brilliant Blue R-250. Protein contents were determined using the method of Bradford [24], with bovine serum albumin as a standard. CbbR was stored at -20°C.

### Production of antisera to CbbR

Multiple intradermal injections were applied to immunize a female Californian giant rabbit (3.0 kg) as described by [25]. A fresh CbbR preparation (0.5 ml; 1 mg/ml) was emulsified in one volume of complete Freund adjuvant (Commonwealth Serum Laboratories, Melbourne, Australia). The emulsion was prepared under aseptic conditions and 1.0 ml was initially injected into four sites on the back of the animal. Booster injections were given in the same way 75 days after the primary immunization, except that incomplete Freund adjuvant was used. The immune response was monitored by Western Blotting assays with serum separated from test blood samples (1.0 to 2.0 ml) that were obtained from an ear vein every 15 to 20 days after each immunization.

### Electrophoretic mobility shift assays (EMSA)

DNA fragments containing the four potential *cbb *operon promoter regions were amplified by PCR and simultaneously biotinylated using the biotin 5'-labelled primers (Table 2). DNA-binding assays were performed at 30°C in a final volume of 17 μl containing 12 mM HEPES-NaOH, pH 7.9, 4 mM Tris-HCl, pH 7.9, 1 mM EDTA, 60 mM KCl, 1 mM dithiothreitol, 10% (w/v) glycerol, 5 μg/μl of bovine serum albumin and 2 μg/μl of poly(dI-dC). The indicated amount of CbbR protein (~290 μM) was incubated with the biotin end-labeled target DNA (20 pmol) for 15 min. A 50-fold excess of unlabeled DNA probe was used to challenge the labeled probe. In supershift experiments, a 1:500 dilution of CbbR-specific antiserum was added to the reaction after DNA binding of CbbR and incubated for an additional 15 min. After the binding reactions, samples were loaded onto a low-ionic strength nondenaturing polyacrylamide gel (4.8% [w/v], which had been prerun at a constant current of 200 mA for more than 90 min, and electrophoresed at 150 mA for about 60 min in 0.5× TBE buffer (89 mM Tris base, 89 mM boric acid and 2 mM EDTA). The separated CbbR-DNA complexes were electrophoretically transferred from the gel onto a nylon membrane in semi-dry blotting apparatus (Biometra, Göttingen). CbbR-DNA binding was detected using a streptavidin-horseradish peroxidase conjugate and a chemiluminescent substrate (Pierce) followed by autoradiography.

### Bioinformatic analyses

Metabolic pathways involved in CO_2 _assimilation were retrieved from KEGG http://www.genome.ad.jp/kegg/. Protein sequences derived from known genes involved in CO_2 _assimilation were used as query sequences to search the genome sequence of *A. ferrooxidans *ATCC 23270, using TBlastN and BlastP, respectively, with default parameters. When a prospective candidate gene was identified, its predicted protein sequence was then used to formulate a BlastP http://www.ncbi.nlm.nih.gov search of the nonredundant database at NCBI. Only bidirectional best hits were accepted as evidence for putative orthologs. Candidate genes and their translated proteins were further characterized employing the following bioinformatic tools: ClustalW [26] for primary structure similarity relations, PSI-PRED [27] for secondary structure predictions, Prosite [28] for motif predictions, ProDom [29] and Pfam [30] for domain predictions. Information regarding the organization of genes in *A. ferrooxidans *was obtained from [2]. Logos were generated using the web-based application available at http://weblogo.berkeley.edu/logo.cgi. The height of each letter in bits corresponds to its relative abundance at each position. Promoters of the σ^70^-type and rho-independent transcriptional stops were predicted for operons *cbb1-4 *using the programs BPROM http://www.softberry.com and Transterm [31], respectively.

The organization of gene clusters in facultative and obligate autotrophs involved in the CBB cycle was derived from information available in IMG-JGI http://www.jgi.doe.gov/ and MicrobesOnline http://www.microbesonline.org/, with additional information added for *H. marinus *[18] and *A. ferrooxidans*, *Acidithiobacillus caldus *and *Acidithiobacillus thiooxidans *(this study). The phylogenetic cladogram of these bacteria was constructed from 16 S rRNA sequences obtained from KEGG Orthology K01977 http://www.genome.jp/kegg/ko.html and from GenBank http://www.ncbi.nlm.nih.gov/ for *A. caldus *(GI454888), *A. thiooxidans *(GI454888) and *H. marinus *(GI3882094). 16 S rRNA alignments were carried out using ClustalW and the cladogram was constructed by the NJ method using the program MEGA 4.0 [32]. The robustness of the tree was evaluated by bootstrapping using 1000 replicas. The tree was rooted using the 16 S rRNA of the ε-proteobacterium *Helicobacter pylori*.

## Results, Discussion and Conclusions

### The genome of *A. ferrooxidans *ATCC 23270 encodes CbbR, a LysR-type transcription factor

A gene *cbbR *was predicted in the genome of *A. ferrooxidans *ATCC 23270 (type strain) that potentially encodes a protein with significant amino acid sequence similarity and domain structure to other well-documented CbbRs of the LysR family of transcription factors (Additional file 1). *cbbR *is divergently transcribed from *cbbL1*, a gene predicted to encode the large subunit of form I RubisCO. The genetic linkage between *cbbR *and *cbbL1 *is known to be conserved in a number of autotrophic bacteria that fix CO_2 _via the CBB cycle such as *Acidithiobacillus ferrooxidans *Fe1 [4], *Hydrogenophilus thermoluteolus *[33], *Nitrosomonas europaea *[19], *Rhodobacter sphaeroides *[34], *Rhodobacter capsulatus *[35], *R. eutropha *H16 [36], *Rhodospirillum rubrum *[17], *Thiobacillus denitrificans *[14] and *Xanthobacter flavus *[9]. We here extend this list to include: *Alkalilimnicola ehrlichii, Halorhodospira halophila*, *Methylibium petroleiphilum, Nitrobacter winogradskyi*, *Nitrosococcus oceani*, *Nitrosospira multiformis, Thiomicrospira crunogena *and *Xanthobacter autotrophicus *(Additional file 2).

**Figure 1:**
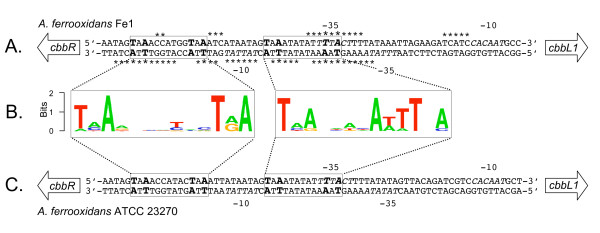
**The *cbbR*-*cbbL1 *intergenic regions of *A. ferrooxidans *strains Fe1 and ATCC 23270**. (A) DNA sequence of *cbbR*-*cbbL1 *intergenic region of *A. ferrooxidans *Fe1 showing two TNA-N_7_-TNA CbbR-binding regions (boxed sequences) and experimentally verified nucleotides protected by CbbR binding (*) and σ^70 ^promoter regions (-10 and -35 sites) (Modified from [5], with permission of the publisher). (B) Logos derived from multiple sequence alignments of the *cbbR*-*cbbL1 *intergenic region of eight bacteria showing conservation of the CbbR-binding sites (more information in additional file 3). (C) Prediction of CbbR-binding sites and σ^70 ^promoter regions in the *cbbR*-*cbbL1 *intergenic region of *A. ferrooxidans *ATCC 23270 by comparison with experimentally verified regions of *A. ferrooxidans *Fe1 and using the information derived from Logos.

The *cbbR*-*cbbL1 *intergenic region of *A. ferrooxidans *strain Fe1 has been shown to contain divergent σ^70^-type promoters and to exhibit two CbbR binding sites that partially overlap these promoters ([4], Figure 1A). The binding sites conform to the pseudo-palindromic motif TNA-N_7_-TNA [13] that is a subset of the consensus LysR-type transcription factor binding site T-N_11_-A [37]. Logos were derived from a multigenome comparison of the *cbbR*-*cbbL1 *intergenic region of a number of bacteria (Additional file 3) and were aligned with the CbbR sites of *A. ferrooxidans *strain Fe1, allowing the prediction of the CbbR binding sites of *A. ferrooxidans *ATCC 27230 (Figure 1B and 1C).

### Organization and expression of gene clusters predicted to be involved in CO_2 _fixation and associated pathways of central carbon metabolism

**Figure 2:**
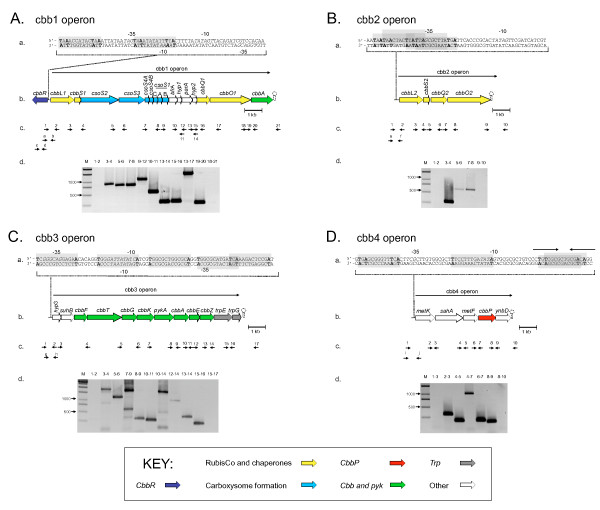
**Organization and co-transcription of four *cbb *gene clusters *in A. ferrooxidans *ATCC 23270**. (A) *cbb1 *(B) *cbb2 *(C) *cbb3 *and (D) *cbb4*. The following are represented in each of the panels A-E: (a) nucleotide sequences of the predicted σ^70^-like promoter region (-10 and -35 sites in italics) and potential CbbR-binding sites in grey boxes with the LysR-type TNA-N_7_-TNA and T-N_11_-A consensus binding sites in bold letters, (b) gene organization of the respective operons with predicted rho-independent transcriptional stop sites indicated as stem-loop symbols, (c) locations of PCR primers used for RT-PCR experiments (indicated by numbers) or EMSA assays (indicated by letters) and (d) gel electrophoresis of fragments amplified by RT-PCR using purified cellular RNA as template. A 1-kb scale bar is shown. One of the T-N_11_-A consensus binding sites in the *cbb4 *operon is part of a larger pseudo-palindrome indicated by inverted arrows. Predicted gene functions are provided in Table 3.

A cluster of 16 genes, termed *cbb1*, was predicted to be involved CO_2 _fixation. RT-PCR experiments showed that *cbb1 *is transcribed as a single unit and thus can be considered to be an operon (Figure 2A). Operon *cbb1 *consists of *cbbL1 *and *cbbS1*, potentially encoding the large and small subunits of form IAc RubisCO, seven *cso *genes predicted to be involved in α-carboxysome formation, two genes (*cbbQ1 *and *cbbO1*) presumed to be involved in RubisCO activation and *cbbA*, potentially encoding a fructose-1,6-bisphosphate aldolase. Gene descriptions are provided in Table 3.

**Table 3:** Predicted genes of *cbb *operons

*Accession	^a^Gene name	^b^Predicted function	^c^Best BlastP hit	^d^% Similarity	^e^Score	^f^E-value	^g^Domains and motifs
**Operon *cbb1***							
ACK78724.1	*cbbR*	LysR family transcriptional regulatory protein CbbR	*Nitrococcus mobilis*	76	363	7e-99	PD462572, PD756396, Pfam03466, Pfam00126, COG0583
ACK79627.1	*cbbL1*	Ribulose bisphosphate carboxylase large subunit 1 [4.1.1.39]	*Halothiobacillus neapolitanus*	94	882	0	PD417314, PD000044, Pfam00016, Pfam02788, COG1850
ACK77836.1	*cbbS1*	Ribulose bisphosphate carboxylase small subunit 1 [4.1.1.39]	*Methylococcus capsulatus*	80	161	8e-39	PD000290, Pfam00101, COG4451
ACK78689.1	*csoS2*	Carboxysome structural peptide	*Thiobacillus denitrificans*	59	325	9e-87	PD579361, tat signal peptide
ACK80925.1	*csoS3*	Carboxysome structural peptide	*Thiobacillus denitrificans*	65	537	5e-151	PD191834, Pfam08936
ACK80352.1	*csoS4A*	Carboxysome peptide A	*Thiobacillus denitrificans*	93	139	6e-32	PD012510, Pfam03319, COG4576, tat signal peptide
ACK79436.1	*csoS4B*	Carboxysome peptide B	*Thiobacillus denitrificans*	82	119	7e-26	PD012510, Pfam03319, COG4576
ACK78722.1	*csoS1C*	Microcompartments protein	*Nitrosomonas eutropha*	97	142	6e-33	PD003442, Pfam00936, COG4577
ACK79154.1	*csoS1A*	Microcompartments protein	*Nitrosomonas eutropha*	97	144	1e-33	PD003442, Pfam00936, COG4577
ACK79584.1	*csoS1B*	Microcompartments protein	*Nitrosomonas eutropha*	95	146	3e-34	PD003442, Pfam00936, COG4577
ACK79096.1	*bfrA*	Bacterioferritin	*Thiobacillus denitrificans*	70	135	6e-31	PDA00179, Pfam00210, COG1633
ACK77923.1	*hyp1*	Hypothetical protein	*Thiobacillus denitrificans*	81	68	2e-10	PDA1E0I5
ACK80576.1	*parA*	Partition protein A	*Thiobacillus denitrificans*	72	196	6e-49	PD194671, Pfam01656, COG1192
ACK78664.1	*hyp2*	Hypothetical protein	*Acidithiobacillus ferrooxidans*	100	156	1e-09	
ACK80060.1	*cbbQ1*	Rubisco activation protein	*Nitrosomonas europaea*	92	489	5e-137	PD490543, Pfam08406, Pfam07728, COG0714, COG5271
ACK80817.1	*cbbO1*	Rubisco activation protein	*Thiobacillus denitrificans*	74	940	0	PD140693, PD679436, Pfam00092, COG4867, COG4548
ACK80290.1	*cbbA*	Fructose-bisphosphate aldolase [4.1.2.13]	*Bradyrhizobium *sp.	61	295	3e-78	PD002376, PD030418, Pfam01116, Pfam07876, COG191
**Operon *cbb2***							
ACK80366.1	*cbbL2*	Ribulose bisphosphate carboxylase/oxygenase large subunit 2 [4.1.1.39]	*Thiobacillus denitrificans*	97	920	0	PD417314, PD000044, Pfam00016, Pfam02788, COG1850
ACK79774.1	*cbbS2*	Ribulose bisphosphate carboxylase/oxygenase small subunit 2 [4.1.1.39]	*Thiobacillus denitrificans*	88	203	3e-51	PD000290, Pfam00101, COG4451
ACK80953.1	*cbbQ2*	Rubisco activation protein	*Nitrosomonas europaea*	92	483	6e-135	PD490543, PD372819; Pfam08406, Pfam07728, COG0714
ACK78928.1	*cbbO2*	Rubisco activation protein	*Thiobacillus denitrificans*	76	965	0	PD140693, PD025507, COG4548
**Operon *cbb3***							
ACK80740.1	*hyp3*	Hypothetical protein	*Thiobacillus denitrificans*	49	149	8e-9	PD796582
ACK78212.1	*suhB*	Inositol-phosphate phosphatase [3.1.3.25]	*Methylococcus capsulatus*	66	646	8e-66	PD001491, PD013702, pfam00459, pfam00316, COG0483, COG1218
ACK80404.1	*cbbF*	Fructose-1,6-bisphosphatase [3.1.3.11]	*Mariprofundus ferrooxydans*	71	823	3e-86	PD007014, PD863173, pfam03320, COG1494
ACK79091.1	*cbbT*	Transketolase [2.2.1.1]	*Methylococcus capsulatus*	75	2264	0.0	PD308336, pfam00456, pfam02779, COG3959, COG0021
ACK78716.1	*cbbG*	Glyceraldehyde-3-phosphate dehydrogenase type I [1.2.1.-]	*Burkholderia thailandensis*	82	1189	1e-128	PD959395, PD859695, pfam02800, pfam00044, COG0057
ACK79414.1	*cbbK*	Phosphoglycerate kinase [2.7.2.3]	*Alcanivorax borkumensis*	80	1296	6e-141	PD000619, PDA014E1, pfam00162, COG0126
ACK78522.1	*pykA*	Pyruvate kinase II [2.7.1.40]	*Thiobacillus denitrificans*	79	1491	2e-163	PD983049, PD745602, pfam00224, pfam02887, COG0469
ACK79923.1	*cbbA*	Fructose-bisphosphate aldolase [4.1.2.13]	*Nitrosococcus oceani*	90	1474	1e-161	PD875785, PD002376, pfam01116, COG0191
ACK80630.1	*cbbE*	Ribulose-5-phosphate 3-epimerase [5.1.3.1]	*Herminiimonas arsenicoxydans*	80	753	2e-78	PD003683, PD591639, pfam00834, COG0036
ACK80633.1	*cbbZ*	Phosphoglycolate phosphatase [3.1.3.18]	*Thiobacillus denitrificans*	64	484	4e-47	PD946755, PDA11895, pfam00702, COG0546, COG0637
ACK78314.1	*trpE*	Anthranilate synthase component I [4.1.3.27]	*Methylococcus capsulatus*	77	1569	2e-172	PD005777, PD105823, pfam00425, pfam04715, COG0147, COG1169
ACK78895.1	*trpG*	Anthranilate synthase component II [4.1.3.27]	*Nitrosomonas europaea*	86	770	2e-80	PD806135, PD976090, pfam00117, pfam07722, COG0512, COG0518
**Operon *cbb4***							
ACK79981.1	*metK*	S-adenosylmethionine synthetase [2.5.1.6]	*Ralstonia eutropha*	86	591	2e-167	PD499406, PD606972, pfam02773, pfam02772, COG0192
ACK78713.1	*sahA*	S-adenosyl-L-homocysteine hydrolase [3.3.1.1]	*Pseudomonas stutzeri*	88	748	0	PD730548, PD551162, pfam05221, pfam00670, COG0499
ACK78001.1	*metF*	5,10-methylenetetrahydrofolate reductase [1.7.99.5]	*Methylococcus capsulatus*	69	306	1e-81	PD756524, PD763008, pfam02219, COG0685
ACK78673.1	*cbbP*	Phosphoribulokinase [2.7.1.19]	*Nitrosococcus oceani*	78	402	2e-110	PD739884, PD015803, pfam00485, COG3954
ACK79243.1	*ynbD*	Phosphosterase, PA-phosphatase	*Polaromonas naphthalenivorans*	81	759	1e-81	PD589889, pfam 01569, COG0474, CD03386, CD00127

* The sequence and annotation of the complete *A. ferrooxidans *strain ATCC 23270 genome is available at the Comprehensive Microbial Resource (CMR) (J. Craig Venter Institute, http://www.jcvi.org) and in GenBank/EMBL/DDBJ accession number CP001219.

^a ^Proposed gene name.

^b ^Proposed enzyme activity with EC number if available

^c ^Organism with the best BlastP hit to the candidate gene.

^d ^Percentage of similarity (% Sim) of candidate gene to that found in the organism listed in row (c).

^e ^Score of BlastP match.

^f ^E value of BlastP match.

^g ^Motif and domains identified in the candidate proteins: CD, Conserved Domains; COG, Clusters of Orthologous Groups of Proteins; Pfam, protein families; PD, Prodom (protein domains); PS, Prosite tat signal peptide

Three additional gene clusters termed *cbb2 *(four genes), *cbb3 *(twelve genes) and *cbb4 *(five genes) were identified that are predicted to encode functions related to CO_2 _fixation and central carbon metabolism (Table 3). RT-PCR experiments revealed that gene clusters *cbb2*, *cbb3 *and *cbb4 *are transcribed as single units, respectively, and thus constitute operons (Figure 2B-D). *cbb2 *contains genes (*cbbL*2 and *cbbS2*) encoding additional copies of the large and small subunit of form IAq RubisCO and associated RubisCO activation genes (*cbbQ2 *and *cbbO2*) (Figure 2B). The deduced amino acid sequences of these genes are similar but not identical to the corresponding proteins encoded in the *cbb1 *operon; CbbL1 and CbbL2 exhibit 84% amino acid sequence identity, whereas CbbS1 and CbbS2 share 56% identity and CbbQ1 and CbbO1 have 84% and 59% identity with CbbQ2 and CbbO2, respectively.

Genes predicted to be encoded by operons *cbb3 *and *cbb4 *are listed in Table 3 and their organization within these operons is shown in Figure 2.

The two enzymes that are unique to the CBB cycle are RubisCO (encoded by operons *cbb1 *and *cbb2*) and phosphoribulokinase (encoded by operon *cbb4*). RuBisCO catalyzes the first step of the cycle, the carboxylation of ribulose-1,5-bisphosphate (RuBP) with CO_2_. Phosphoribulokinase catalyzes the last step of the cycle which is the regeneration of the CO_2 _acceptor molecule, RuBP, by phosphorylation of ribulose 5-phosphate with ATP. Other steps of the cycle, encoded in operon *cbb3*, are catalyzed by enzymes common to glycolytic and gluconeogenic pathways in central carbon metabolism [8,36].

Promoters of the σ^70^-type and rho-independent transcriptional stops were predicted for operons *cbb1-4 *(Figure 2). In addition, potential CbbR-binding sites were identified in the four operons based on the detection of conserved TNA-N_7_-TNA and T-N_11_-A motifs described above for operon *cbb1 *(Figure 2).

### CbbR binds *in vitro *to the predicted σ^70^-like promoter regions of operons *cbb1-4*

Binding of CbbR to DNA fragments containing the predicted promoters of the four operons *cbb1-4 *was evaluated *in vitro *by electrophoretic mobility shift assays (EMSAs). For this purpose the *cbbR *gene was cloned and expressed in *E. coli*. Purified CbbR was used to prepare antisera (anti-CbbR antibodies) whose activity was checked by Western blotting against purified CbbR (data not shown). Biotin-labeled promoter DNA for the EMSA assays was prepared by PCR using primers specified in Table 2 and whose locations within the four operons are shown in Figure. 2.

**Figure 3:**
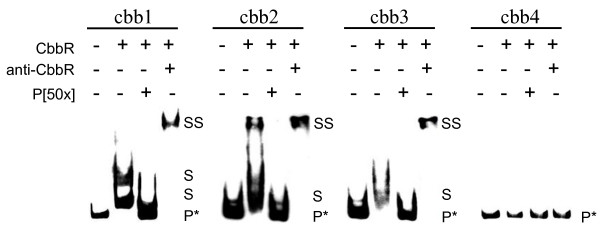
**Binding of CbbR to the promoter regions of the operons *cbb1-4 *using the EMSA assay in the presence (+) or absence (-) of competing 50× excess of unlabelled probe DNA (P[50x]) or antibodies to CbbR (anti-CbbR)**. Abbreviations: P*, probe DNA; S, shift; SS, supershift.

Results show that CbbR was able to retard the promoter regions of the *cbb1*, *cbb2 *and *cbb3 *operons but not the *cbb4 *operon (Figure 3). When a 50-fold molar excess of unlabelled fragment was included in the binding assay retardation of the labelled fragments was abolished. Furthermore, the addition of anti-CbbR antibodies to the reaction produced a supershift in migration, indicating that the shift was caused specifically by the binding of CbbR.

Binding of CbbR to the predicted promoter regions of operons *cbb1-3 *suggests that it is involved in their regulation. The reason for the failure of CbbR to retard the DNA fragment containing the predicted promoter of the *cbb4 *operon is not known. Perhaps this fragment requires the presence of additional factors for CbbR binding that are not present in the *in vitro *cocktail used for the EMSA analysis. Alternatively, the predicted CbbR binding site is not functional.

### Gene organization of the *cbb *operons

The *cbb3 *operon includes not only genes involved in carbon assimilation but also harbors genes with similarity to *trpE *and *trpG *that are predicted to encode the components I and II of anthranilate synthase, the first enzyme of the tryptophan biosynthesis pathway. Anthranilate synthase catalyzes the conversion of chorismate to anthranilate with the concomitant release of pyruvate [38,39]. In some cases, this conversion can be accomplished by TrpE alone [40].

**Figure 4:**
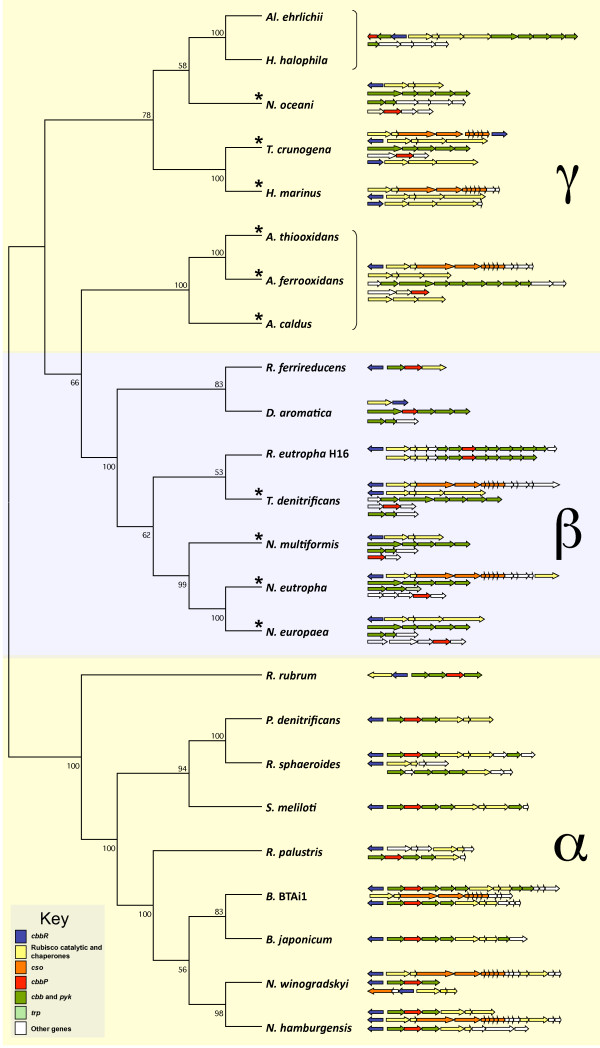
**Organization of gene clusters involved in the CBB cycle of facultative and obligate autotrophic α-, β- and γ-proteobacteria presented as a phylogenetic cladogram based on 16 S RNA**. Numbers refer to bootstrapping results from 1000 trees. Organism names are provided in the text. The asterisk indicates that the respective organism is an obligate autotroph.

In order to determine if the association between *trpEG *and the *cbb *genes is restricted to *A. ferrooxidans*, an examination of gene organization was carried out in all sequenced genomes of facultative and obligate autotrophic proteobacteria. Twenty-six proteobacterial organisms (11 α-, 7 β- and 8 γ-) were analyzed, including 10 obligate autotrophs. Linkage between *trpE/G *and *cbbE *and/or *cbbZ *was found in all sequenced obligate autotrophs, all of which belong to the β- or γ-proteobacteria divisions (Figure 4, Table 4), whereas only 4 out of 14 facultative heterotrophs were detected with this clustering. These four exceptions are found only in the β- or γ-proteobacteria and none in the α-proteobacterial division (Figure 4, Table 4). This suggests a previously unreported linkage between genes encoding CBB cycle associated enzymes and *trpEG *or *trpE *that is most conserved in obligate autotrophs of the β- and γ-proteobacteria.

**Table 4:** Characteristics of *cbb *gene clusters in facultative and obligate, autotrophic bacteria.

Organism	Autotrophy status	Phyogenetic classification -proteo-bacteria	No. copies *cbbR*	*Presence* *of cso *genes?	*trpE/G* *associated *with *cbb*?	*cbb *gene cluster associated with *cbbP*?	No. *cbb* gene clusters
*Acidithiobacillus ferrooxidans ATCC 23270 and ATCC 53993*	obligate	Gamma-	2	Yes	Yes	No	5*

*Acidithiobacillus thiooxidans ATCC 19377*	obligate	Gamma-	2	Yes	Yes	No	5

*Acidithiobacillus caldus ATCC 51756*	obligate	Gamma-	2	Yes	Yes	No	5

*Nitrosomonas europaea *ATCC 19718	obligate	Beta-	1	No	Yes	No	4

*Nitrosomonas eutropha C71*	obligate	Beta-	1	Yes	Yes	No	4

*Nitrosococcus oceani *ATCC 19707	obligate	Beta-	1	No	Yes	No	4

*Thiomicrospira crunogena *XCL-2	obligate	Gamma-	3	Yes	Yes	No	5

*^5^Hydrogenovibrio marinus MH-110*	obligate	Gamma-	2	Yes	N/D	N/D	3

*Thiobacillus denitrificans *ATCC 25259	obligate	Beta-	2	Yes	Yes	No	5

*Nitrosospira multiformis *ATCC 25196	obligate	Beta-	1	No	Yes	No	4

*Methylococcus capsulatus *Bath	obligate methanotroph	Gamma-	1	No	Yes	Yes	3

*^1^Nitrobacter hamburgensis *X14	facultative	Alpha-	3	Yes	No	Yes	3

*Nitrobacter winogradskyi *Nb-255	facultative	Alpha-	3	Yes	No	Yes	3

*Halorhodospira halophila *SL1	facultative	Gamma-	1	No	Yes^3^	Yes	2

*Alkalilimnicola ehrlichii *MLHE-1	facultative	Gamma-	1	No	Yes^3^	Yes	2

*Bradyrhizobium sp. BTAi1*	facultative	Alpha-	2	Yes	No	Yes	3

*Bradyrhizobium japonicum *USDA 110	facultative	Alpha-	1	No	No	Yes	1

*Ralstonia eutropha *H16	facultative	Beta-	1	No	No	Yes	2^4^

*Dechloromonas aromatica *RCB	facultative	Alpha-	1	No	No	Yes	2

*^2^Magnetospirillum magneticum AMB-1*	facultative	Alpha-	?	No	No	Yes	2

*Paracoccus denitrificans *PD1222	facultative	Alpha-	1	No	No	Yes	1

*Rhodobacter sphaeroides *2.4.1	facultative	Alpha-	1	No	No	Yes	2

*Rhodoferax ferrireducens *T118	facultative	Beta-	1	No	No	Yes	1

*Rhodopseudomonas palustris *CGA009	facultative	Alpha-	2	No	No	Yes	3

*Rhodospirillum rubrum *ATCC 11170	facultative	Alpha-	1	No	No	Yes	1

*Sinorhizobium meliloti *1021	facultative	Alpha-	1	No	No	Yes	1

*in addition to the four *cbb *operons described in this paper, a fifth gene cluster containing *cbb *genes (including a form II RubisCO gene) has recently been detected in *A. ferrooxidans *(43). ^1^Two copies of *cbbR *and two *cbb *gene clusters are present on two plasmids; ^2^two highly similar operons present in the genome; ^3^in these organisms, *trpE *gene is neighbor to *cbbP *but not *cbbE*. ^4 ^*R. eutropha *H16 posesses a duplicated *cbb *operon, with similar copies in the chromosome and in a megaplasmid. *^5^*Data derived from cloned sequences (18). N/D = no data.

We hypothesize that in *A. ferrooxidans *production of pyruvate via anthranilate synthase activity provides a novel network connection between the CBB cycle on the one hand and general central carbon metabolism including the incomplete ("horseshoe"-like) TCA [2] on the other hand. Consistent with this idea is the presence of a predicted *pykA *upstream of *trpEG *in the *cbb3 *operon. *PykA *is predicted to encode pyruvate kinase that catalyzes the conversion of phosphoenol pyruvate (PEP) to pyruvate. In addition to supplying pyruvate, PykA could also reduce the level of intracellular PEP. PEP has been shown to be a ligand of CbbR in *Ralstonia eutropha *H16, promoting its binding to target DNA sites and consequently effecting the regulation of *cbb *genes [40]. If PEP carries out a similar function in *A. ferrooxidans*, the depletion of PEP via PykA activity could provide a means for feedback control of operons that are regulated by CbbR, including the auto-regulation of operon *cbb3*.

The organization of *cbb *genes in *A. ferrooxidans *exhibits similarities with obligate autotrophs that distinguish this group from facultative autotrophs. For example, *A. ferrooxidans*, contains three or more gene clusters dedicated to carbon assimilation. This is similar to other obligate autotrophic γ-proteobacteria including *A. caldus*, *A. thiooxidans*, *Hydrogenovibrio marinus*, *Nitrosococcus oceani and Thiomicrospira crunogena*, and obligate autotrophic β-proteobacteria such as *Nitrosomonas europaea, Nitrosomonas eutropha, and Nitrosospira multiformis *and *Thiobacillus denitrificans*. This contrasts with facultative autotrophs that contain only one or two *cbb *clusters (Figure 4, Table 4), with some exceptions, e.g. the α-proteobacteria *Bradyrhizobium sp*., *N. hamburgensis*, *N. winogradski. R. sphaeroides *and *R. palustris *and the β-proteobacterium *R. eutropha*, which contain unique, but duplicated, *cbb *clusters). Multiple *cbb *clusters could provide obligate autotrophs with a greater flexibility in regulating CO_2 _fixation compared to facultative autotrophs. For example, this flexibility may be necessary to adjust carbon assimilation in response to changing environmental concentrations of CO_2 _[18], whereas facultative autotrophs might be able to circumvent this need by exploiting organic carbon sources in times of low CO_2 _concentrations.

Another characteristic of *cbb *gene organization in *A. ferrooxidans *is the lack of linkage of the phosphoribulokinsae gene, *cbbP*, with other *cbb *genes (Figure 4, Table 4) as has previously been reported for the deep-sea vent obligate chemolithoautotroph *T. crunogena *XCL-2 and for several other obligate autotrophs [20,41]; we now extend this list to include *A. ferrooxidans *ATCC 23270 and ATCC 53993, *A. caldus*, *A. thiooxidans H. marinus*, *N. europaea *and *Thiomicrospira crunogena *(Figure 4, Table 4). In contrast, in all sequenced facultative autotrophs *cbbP *is associated with other *cbb *genes (Figure 4, Table 4).

In obligate autotrophs, the contextual disconnection of *cbbP *from *cbbLS *could provide greater flexibility for CO_2 _fixation by allowing RubisCO to be differentially expressed according to environmental and/or metabolic requirements without simultaneously expressing the remaining CBB cycle genes, many of which carry out functions in addition to carbon fixation. This is in sharp contrast to the organization found in most facultative autotrophs, where *cbb*P is usually juxtaposed to *cbbLS *and other genes of the CBB cycle facilitating their coordinate repression during heterotrophic growth [13,20,34,36,41].

### Model for predicted enzymes and pathways involved in CO_2 _fixation

**Figure 5:**
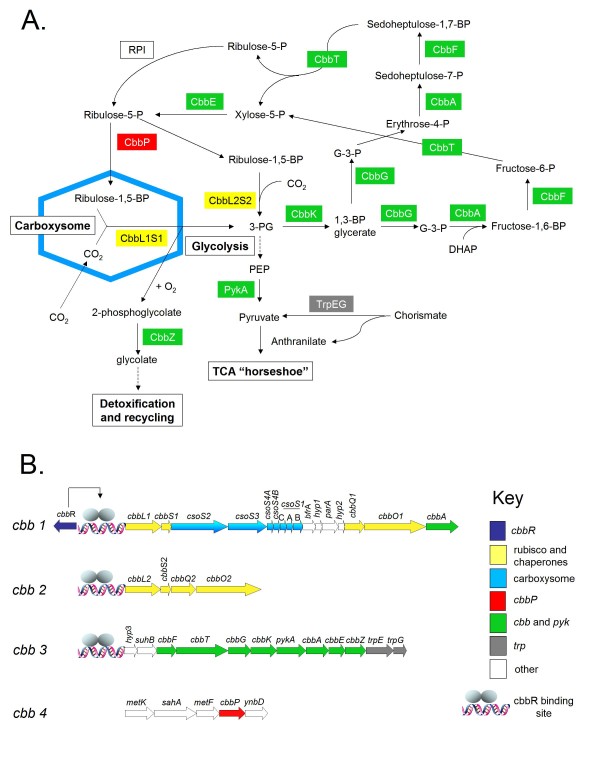
**Proposed roles of the (A) predicted enzymes and pathways involved in CO_2 _fixation in *A. ferrooxidans *linked to (B) gene evidence**. Genes are color-coded to match the predicted function of their products. RPI, ribose phosphate isomerase; G-3-P, glyceraldehyde-3-phosphate; DHAP, dihydroxyacetone phosphate; 3-PG, 3-phosphoglycerate; PEP, phosphoenolpyruvate.

A model is proposed for C_i _fixation in *A. ferrooxidans *based on the predicted roles of the genes encoded in the *cbb *operons (Figure 5). In contrast to most facultative autotrophs, the main focus of regulation of the CBB cycle in *A. ferrooxidans *may be the CO_2 _fixation reaction itself catalyzed by RubisCO, rather than at the level of the other CBB cycle enzymes. This hypothesis is supported by the observation that the genes encoding RubisCO and RubisCo accessory proteins, are clustered in operons that do not contain *cbbP *nor *cbb *that encode the main CBB enzymes. *cbbP *is also separated from the rest of the *cbb *genes in the *cbb4 *operon, with an apparent absence of CbbR binding to its promoter. We suggest that the promoters for the *cbb1*, *cbb2 *and *cbb3 *operons have different affinities for CbbR and may thus exhibit different regulation patterns, possibly associated with the environmental availability of CO_2_. The *cbb1 *operon, containing *cbbLS-cso*, is predicted to serve at low CO_2 _concentrations because carboxysomes have been shown to improve RubisCO catalytic efficiency by concentrating CO_2 _[6,13]. In contrast, the *cbb2 *operon, containing *cbbLSQO*, is predicted to be used when higher concentrations of CO_2 _are available since carboxysome synthesis is energetically and materially expensive [18].

The *cbb3 *operon, containing genes for most CBB cycle enzymes and pyruvate kinase, is proposed to be responsible for connecting CO_2 _fixation with the rest of central carbon metabolism. Except for *cbbG *and *cbbK *encoding glyceraldehyde-3-phosphate dehydrogenase, type I and phosphoglycerate kinase respectively, genes of the *cbb3 *operon have duplicated copies in the genome (data not shown), potentially allowing regulation of the CBB cycle independently of the remaining pathways of central carbon metabolism. For example, some CBB cycle intermediates also form part of gluconeogenesis and glycolysis resulting in the production of pyruvate that is channeled, via the pyruvate dehydrogenase complex, into the incomplete TCA "horseshoe" where the flux of intermediates serves for amino acid biosynthesis (e.g. glutamate). The pyruvate dehydrogenase also provides acetyl-CoA used in fatty acid biosynthesis. In addition, the presence of *cbbZ *in the *cbb3 *operon is associated with phosphoglycolate phosphatase activity, responsible for removal of phosphoglycolate, an undesirable product of the oxygenase activity of RubisCO, that must be detoxified preferentially by rechanneling to 3-phosphoglycerate [13,36].

The co-transcriptional connection between the *cbb*, *pykA *and *trpEG *genes in the *cbb3 *operon may reflect the substrate requirement of anthranilate phosphoribosyltransferase for an activated pentose (5-phosphoribosyl 1-pyrophosphate) in order to proceed to the next step of tryptophan biosynthesis [42]. The production of the activated pentose would be stimulated by the activity of the operon. An alternate hypothesis is that the co-transcriptional connection represents a means for pyruvate regeneration since both *pykA *and *trpE/G *produce pyruvate.

In addition to the four *cbb *operons described herein, a fifth gene cluster has recently been detected in *A. ferrooxidans *that includes genes *cbbM*, *cbbQ3 *and *cbbO3 *predicted to encode form II of RubisCO and its associated chaperons, respectively [43]. The cluster also contains another putative *cbbR *divergently transcribed from *cbbMQO*. Future work will evaluate the role of this cluster in CO_2 _fixation.

## Authors' contributions

DH, EJ and ME conceived the study. ME carried out the experiments. BB and J-PC contributed significantly to the analysis and interpretation of results. DH drafted the manuscript. All authors contributed to the draft and approved the manuscript.

## Supplementary Material

Additional file 1 **Prediction of secondary structure elements in CbbR of *Acidithiobaillus ferrooxidans***. Above: secondary structure predictions of alpha-helix, beta-sheet, HTH DNA binding domain, oligomerization domain and LysR-substrate like domain. Below: alignment of amino acid sequences from the HTH domain from several bacteria (abbreviations used can be found in Additional File 2) with the pfam domain00126. Click here for file

Additional file 2 **Alignment and conservation of DNA sequences in the intergenic regions between *cbbR *and *cbbL1 *in autotrophic bacteria**. The DNA sequences contain the cbb control elements including the operator, the operon promoter (p*cbbL*) and the promoter cbbR (p*cbbR*). The CbbR regulator bind to region R (recognition site) and the region A (activation site) of the cbb operator. The nucleotides conserved (TNA-N_7/8_-TNA, T-N_11_-A) for to bind CbbR are located in intergenic regions RI-1, RI-2 and RI-3. The prediction of the promoter and the sites for to bind σ70 are in the columns (sequences -35 and -10). The names of bacterias are: *Acidithiobacillus ferrooxidans *(Af), *Hydrogenophilus thermoluteolus *(Ht), *Xanthobacter flavus *(Xf), *Nitrosomonas europea *(Ne), *Rhodobacter capsulatus *(Rc), *Rhodobacter sphaeroides *(Rs), *Ralstonia eutropha *H16 (Ral), *Ralstonia metallidurans *CH34 (Rm), *Rhodospirillum rubrum *(Rr), *Nitrococcus oceani *(No), *Nitrobacter winogradskyi *(Nw), *Halorhodospira halophila *(Hh), *Xanthobacter autotrophicus *(Xa)*, Thiomicrospira crunogena *(Tc), *Methylibium petroleiphilum *(Mp)*, Thiobacillus denitrificans *(Td)*, Nitrosospira multiformes *(Nm)*, Alkalilimnicola ehrlichii *(Ae). I and II indicated cbbI and cbbII operons. Af23270 type strain from *A. ferrooxidans*. Af Fe1 strain from Kusano and Sugawara (1993)[4]. Click here for file

Additional file 3 **Sequences used to generate LOGOS of the intergenic region between *cbbR *and *cbbL1***. Click here for file
