# Supplementary material for: Genes and pathways for CO2 fixation in the obligate, chemolithoautotrophic acidophile, Acidithiobacillus ferrooxidans, Carbon fixation in A. ferrooxidans
Source: BMC Microbiol. 2010 Aug 27;10:229. doi: 10.1186/1471-2180-10-229 (PMC2942843; doi:10.1186/1471-2180-10-229)
Supplement: Additional file 1 — Prediction of secondary structure elements in CbbR of Acidithiobaillus ferrooxidans. Above: secondary structure predictions of alpha-helix, beta-sheet, HTH DNA binding domain, oligomerization domain and LysR-substrate like domain. Below: alignment of amino acid sequences from the HTH domain from several bacteria (abbreviations used can be found in Additional File 2) with the pfam domain00126. [file 1471-2180-10-229-S1.PDF]

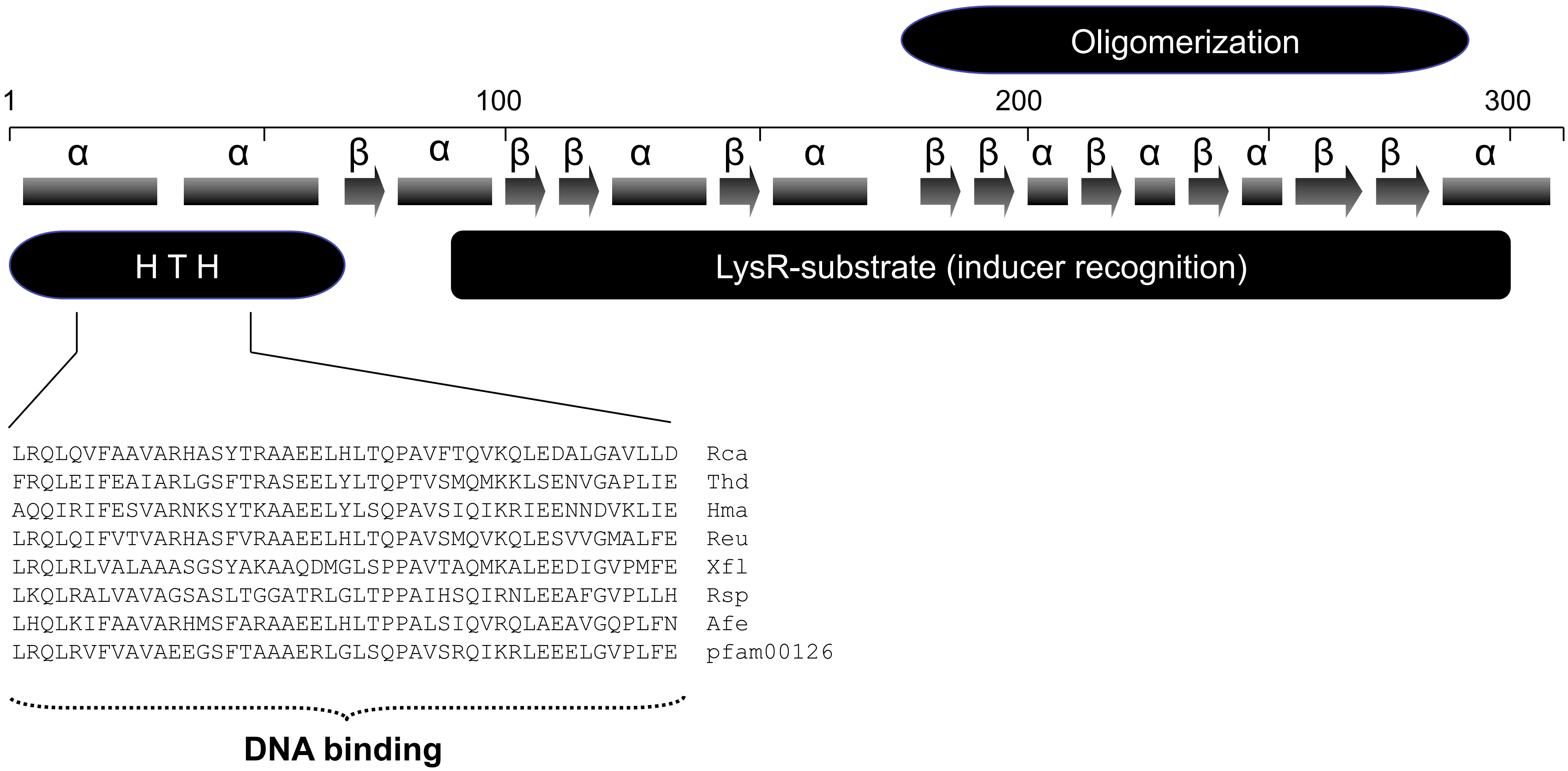
Oligomerization
1
100
200
300
α α β α β β α β α β β α β α β α β β α
H T H
LysR-substrate (inducer recognition)
LRQLQVFAAVARHASYTRAAEELHLTQPAVFTQVKQLEDALGAVLLD Rca
FRQLEIFEAIARLGSFTRASEELYLTQPTVSMQMKKLSENVGAPLIE Thd
AQQIRIFESVARNKSYTKAAEELYLSQPAVSIQIKRIEENNDVKLIE Hma
LRQLQIFVTVARHASFVRAAEELHLTQPAVSMQVKQLESVVGMALFE Reu
LRQLRLVALAAASGSYAKAAQDMGLSPPAVTAQMKALEEDIGVPMFE Xfl
LKQLRALVAVAGSASLTGGATRLGLTPPAIHSQIRNLEEAFGVPLLH Rsp
LHQLKIFAAVARHMSFARAAEELHLTPPALSIQVRQLAEAVGQPLFN Afe
LRQLRVFVAVAEEGSFTAAAERLGLSQPAVSRQIKRLEEELGVPLFE pfam00126
DNA binding
