# Supplementary figures and images for: Genes and pathways for CO2 fixation in the obligate, chemolithoautotrophic acidophile, Acidithiobacillus ferrooxidans, Carbon fixation in A. ferrooxidans
Source: BMC Microbiol. 2010 Aug 27;10:229. doi: 10.1186/1471-2180-10-229 (PMC2942843; doi:10.1186/1471-2180-10-229)

## Additional File 2

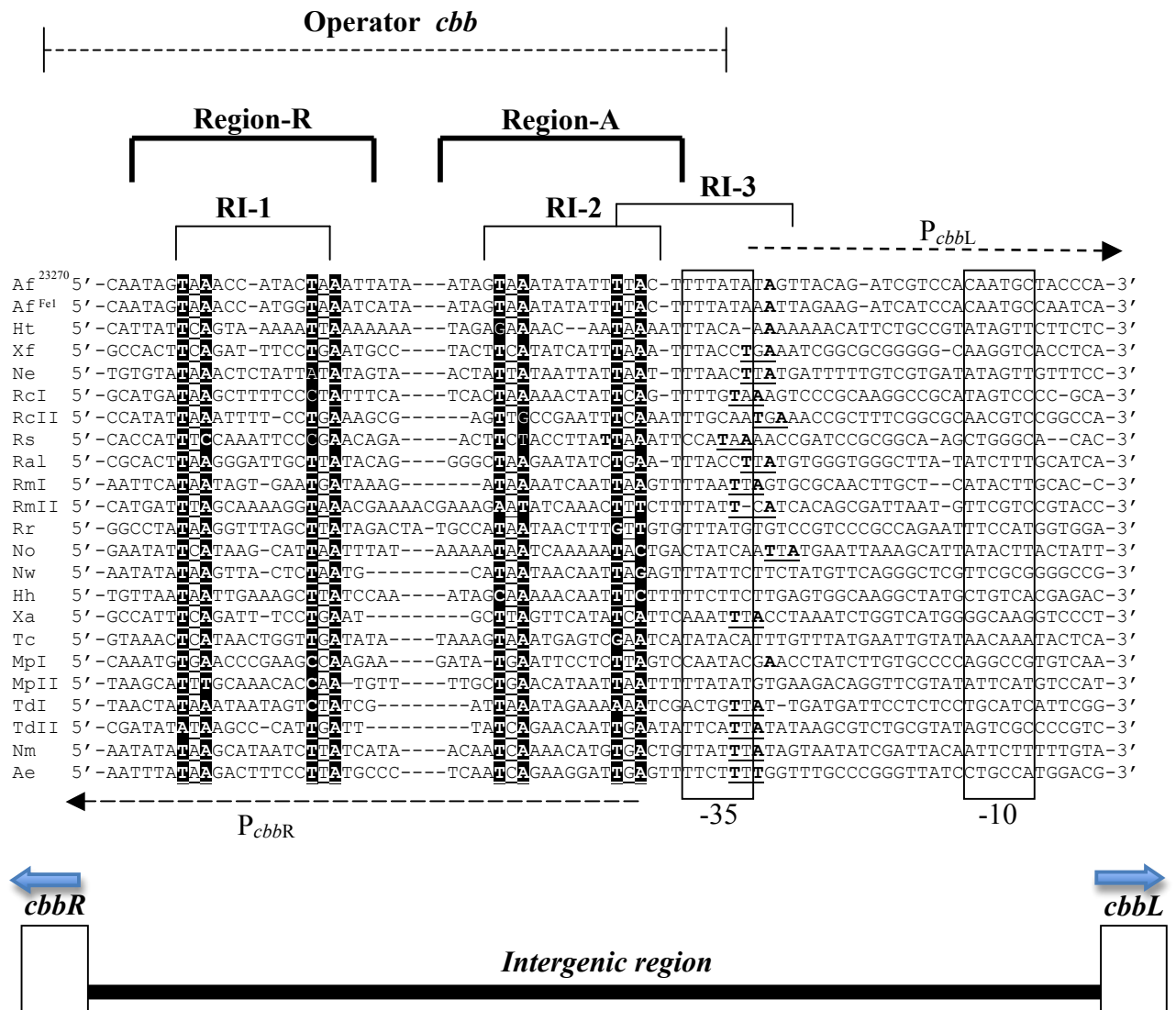

Supplement: Additional file 2 — Alignment and conservation of DNA sequences in the intergenic regions between cbbR and cbbL1 in autotrophic bacteria. The DNA sequences contain the cbb control elements including the operator, the operon promoter (pcbbL) and the promoter cbbR (pcbbR). The CbbR regulator bind to region R (recognition site) and the region A (activation site) of the cbb operator. The nucleotides conserved (TNA-N7/8-TNA, T-N11-A) for to bind CbbR are located in intergenic regions RI-1, RI-2 and RI-3. The prediction of the promoter and the sites for to bind σ70 are in the columns (sequences -35 and -10). The names of bacterias are: Acidithiobacillus ferrooxidans (Af), Hydrogenophilus thermoluteolus (Ht), Xanthobacter flavus (Xf), Nitrosomonas europea (Ne), Rhodobacter capsulatus (Rc), Rhodobacter sphaeroides (Rs), Ralstonia eutropha H16 (Ral), Ralstonia metallidurans CH34 (Rm), Rhodospirillum rubrum (Rr), Nitrococcus oceani (No), Nitrobacter winogradskyi (Nw), Halorhodospira halophila (Hh), Xanthobacter autotrophicus (Xa), Thiomicrospira crunogena (Tc), Methylibium petroleiphilum (Mp), Thiobacillus denitrificans (Td), Nitrosospira multiformes (Nm), Alkalilimnicola ehrlichii (Ae). I and II indicated cbbI and cbbII operons. Af23270 type strain from A. ferrooxidans. Af Fe1 strain from Kusano and Sugawara (1993)[4]. [file 1471-2180-10-229-S2.PDF]
