# Supplementary material for: Genes and pathways for CO2 fixation in the obligate, chemolithoautotrophic acidophile, Acidithiobacillus ferrooxidans, Carbon fixation in A. ferrooxidans
Source: BMC Microbiol. 2010 Aug 27;10:229. doi: 10.1186/1471-2180-10-229 (PMC2942843; doi:10.1186/1471-2180-10-229)
Supplement: Additional file 3 — Sequences used to generate LOGOS of the intergenic region between cbbR and cbbL1. [file 1471-2180-10-229-S3.PDF]

Additional File 3

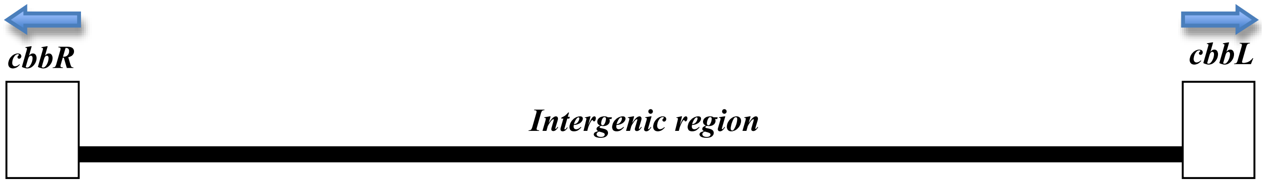


```
AfFe1      5'-CAATAGTAAACCATGGTAAATCATAATAGTAAATATATTTTAC-TTTTATAAATTAGAAG-ATCATCCACAATGCCAA-3'
Af23270    5'-CAATAGTAAACCATACTAAATTATAATAGTAAATATATTTTAC-TTTTATATAGTTACAG-ATCGTCCACAATGCTAC-3'
At         5'-CAATAGTAAACCATACTAAATTATAATAGTAAATATATTTTACTTTTATGGAAGGCACTTTGTACATTCGTCAAGGGT-3'
No         5'-GAATATTCATAAGCATTAATTTATAAAAATAATCAAAAATACTGACTATCAATTATGAATTAAAGCATTATACTTACT-3'
RcII       5'-CCATATTAAATTTTCCTGAAAGCG---AGTTGCCGAATTTCAAATTTGCAATGAAACCGCTTTCGGCGCAACGTCCGG-3'
Rm         5'-AATTCATAATAGTGAATGATAAAG----ATAAAATCAATTAAGTTTTAATTAGTGCGCAACTTGCT--CATACTTGCA-3'
Td         5'-CGATATATAAGCCCATTGATT------TATCAGAACAATTGAATATTCATTATATAAGCGTCTGCGTATAGTCGCCCC-3'
Xf         5'-GCCACTTCAGATTTCCTGAATGCC-TACTTCATATCATTTAAA-TTTACCTGAAATCGGCGCGGGGG-CAAGGTCACC-3'
```

Abbreviation	Organism	GenBank Accession number of *cbbR*
AfFe1	*Acidithiobacillus ferrooxidans* Fe1	BAA01917
Af23270	*Acidithiobacillus ferrooxidans* ATCC 23270	ACK78724.1
At	*Acidithiobacillus thiooxidans*	ACR56721.1
No	*Nitrosococcus oceani* ATCC 19707	YP_342390.1
RcII	*Rhodobacter capsulatus*	AAC32304.1
Rm	*Ralstonia metallidurans* CH34	YP_586499.1
Td	*Thiobacillus denitrificans* ATCC 25259	YP_316383.1
Xf	*Xanthobacter flavus*	CAA80406.1
